# Meeting a Proposed Estimated Glomerular Filtration Rate Slope-Based Criterion for Clinical Remission of Chronic Kidney Disease During Sequential Kidney-Protective Therapy in Type 2 Diabetes Mellitus: A Case Report

**DOI:** 10.3390/jcm15208149

**Published:** 2026-10-20

**Authors:** Tingting Luo, Liwei Wang, Shichun Huang, Zhihong Wang

**Affiliations:** Department of Endocrinology, The First Affiliated Hospital of Chongqing Medical University, Chongqing 400016, China

**Keywords:** chronic kidney disease remission, finerenone, GLP-1RA, SGLT2i, case report

## Abstract

**Background**: A recently proposed estimated glomerular filtration rate (eGFR) slope-based criterion for clinical remission of chronic kidney disease (CKD) represents an emerging therapeutic paradigm. However, clinical courses meeting this criterion after sequential combination therapy with kidney-protective agents have rarely been reported in patients with type 2 diabetes mellitus (T2DM) and CKD. **Case Presentation**: A 70-year-old man with long-standing T2DM, nonproliferative diabetic retinopathy, and progressive albuminuric CKD presented in May 2023 with a urine albumin-to-creatinine ratio (UACR) of 121.60 mg/g creatinine (Cr), an eGFR of 28.00 mL/min/1.73 m^2^, hemoglobin A1c (HbA1c) of 7.5%, and serum potassium of 4.4 mmol/L. Kidney function had continued to decline despite treatment with a renin-angiotensin system inhibitor (RASi), a sodium-glucose cotransporter 2 inhibitor (SGLT2i), and insulin. Therapy was sequentially intensified with a glucagon-like peptide-1 receptor agonist (GLP-1RA) and finerenone, with close monitoring of kidney function and serum potassium. By January 2026, UACR had decreased to 26.53 mg/g Cr and eGFR had increased to 42.48 mL/min/1.73 m^2^. No adverse event required treatment discontinuation. **Results**: Ordinary least-squares regression of all 10 eGFR measurements yielded a positive slope that met the recently proposed eGFR slope-based criterion for clinical remission of CKD. **Conclusions**: In this patient with T2DM and CKD, the eGFR slope over the full follow-up met the proposed criterion for clinical remission of CKD during sequential multidrug kidney-protective therapy with safety monitoring. These observations require confirmation in prospective controlled studies.

## 1. Introduction

Diabetes is a leading cause of chronic kidney disease (CKD) and kidney failure, and CKD substantially increases the risks of kidney failure, cardiovascular events, and premature death in people with diabetes [1]. Management of type 2 diabetes mellitus (T2DM) with CKD has shifted from glycemic control alone toward integrated cardiorenal protection [2]. For patients with T2DM and CKD, current guidelines support layered, risk-based therapy to reduce cardiorenal risk, including maximally tolerated renin-angiotensin system inhibition (RASi) in those with hypertension and albuminuria; sodium-glucose cotransporter 2 inhibition (SGLT2i) when estimated glomerular filtration rate (eGFR) permits; glucagon-like peptide-1 receptor agonist (GLP-1RA) therapy for additional glycemic and cardiorenal benefit; and finerenone, a nonsteroidal mineralocorticoid receptor antagonist (nsMRA), in those with persistent albuminuria and normal serum potassium [2].

Recently, clinical remission has been proposed as a potential therapeutic goal in CKD, beyond slowing disease progression [3]. Tangri et al. systematically proposed the novel concept of CKD remission for the first time in the January 2026 issue of Kidney International, defining it as either a chronic eGFR slope more than −1 mL/min/1.73 m^2^ per year or normalization of eGFR (≥60 mL/min/1.73 m^2^) and albuminuria [3]. It has not yet been validated in prospective cohorts.

Although large trials support kidney protection with all four drug classes, real-world reports using the recently proposed eGFR slope-based criterion to evaluate kidney-function trajectories during sequential combination therapy in patients with T2DM and CKD remain scarce [4,5,6,7]. We describe a patient with T2DM and albuminuric CKD whose full-follow-up eGFR slope met the proposed criterion for clinical remission of CKD during sequential kidney-protective therapy. Urine albumin-to-creatinine ratio (UACR) declined overall, with the last four measurements below 30 mg/g creatinine (Cr).

## 2. Case Presentation

A 70-year-old man first presented to our endocrinology outpatient clinic on 17 May 2023. At presentation, the principal clinical concern was progressive kidney-function decline with persistent albuminuria despite ongoing kidney-protective therapy. He had been diagnosed with T2DM in 2010 after developing thirst, polydipsia, increased appetite, and fatigue, and was initially treated with metformin and glimepiride. Proteinuria was first detected in 2016, after which acarbose was added. In 2017, he was hospitalized with an eGFR of 47.19 mL/min/1.73 m^2^ and two consecutive UACR measurements of 165.7 mg/g Cr and 105.6 mg/g Cr. Renal ultrasonography showed normal-sized kidneys with a left renal cyst. At discharge, his diagnoses included T2DM, CKD G3aA2, and nonproliferative diabetic retinopathy. His discharge medications included acarbose, metformin, glimepiride, irbesartan/hydrochlorothiazide, other antihypertensive agents, and lipid-lowering therapy.

Thereafter, kidney function declined overall. In March 2021, when eGFR was 41.60 mL/min/1.73 m^2^, the glucose-lowering regimen was changed to metformin and insulin glargine. In April 2022, with eGFR of 42.40 mL/min/1.73 m^2^, therapy was revised to insulin degludec/insulin aspart (IDegAsp) and empagliflozin. In April 2023, eGFR was 36.72 mL/min/1.73 m^2^. At presentation to our clinic, he was receiving IDegAsp, empagliflozin, valsartan/amlodipine, and atorvastatin. From April through May 2023, the medical records did not document any acute illness, clinically evident volume depletion, use of diuretics or nonsteroidal anti-inflammatory drugs, contrast exposure, a diagnosis of acute kidney injury (AKI), or treatment directed at AKI.

His medical history included hypertension, hypertensive heart disease, carotid atherosclerosis, dyslipidemia, and prior smoking. He had no known family history of hereditary kidney disease. At the time, body mass index (BMI) was 26.2 kg/m^2^, UACR was 121.6 mg/g Cr, eGFR was 28.00 mL/min/1.73 m^2^, hemoglobin A1c (HbA1c) was 7.5%, and serum potassium was 4.4 mmol/L. No clinically evident volume depletion, peripheral edema, or other signs of volume overload were documented at presentation. Urinalysis was positive for protein and showed 3+ glucose, without hematuria or other reported abnormalities suggestive of active urinary sediment. The clinical diagnosis was T2DM with CKD G4A2.

**Figure 1:**
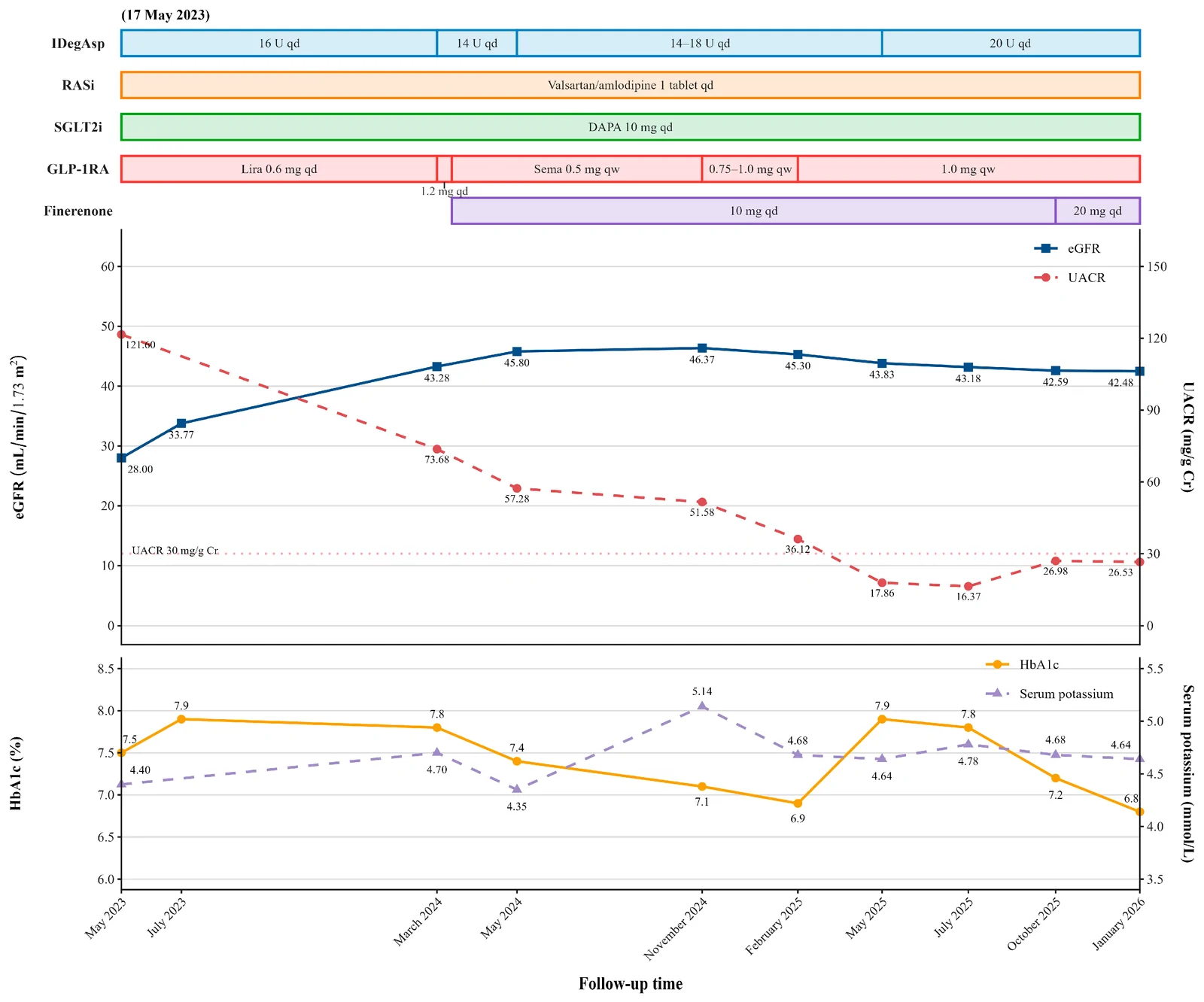
**Clinical Course During Sequential Implementation of Kidney-Protective Therapies.** The upper panel shows the prescribed medications and dose changes from presentation on 17 May 2023, through the final follow-up on 9 January 2026. The middle panel shows the eGFR and UACR trajectories. The lower panel shows HbA1c and serum potassium, which ranged from 6.8% to 7.9% and from 4.35 to 5.14 mmol/L, respectively. At presentation, empagliflozin was discontinued and replaced on the same day with dapagliflozin as a within-class SGLT2i switch, without overlapping use. Abbreviations: Cr, creatinine; DAPA, dapagliflozin; eGFR, estimated glomerular filtration rate; GLP-1RA, glucagon-like peptide-1 receptor agonist; HbA1c, hemoglobin A1c; IDegAsp, insulin degludec/insulin aspart; Lira, liraglutide; qd, once daily; qw, once weekly; RASi, renin-angiotensin system inhibitor; Sema, semaglutide; SGLT2i, sodium-glucose cotransporter 2 inhibitor; U, units; UACR, urine albumin-to-creatinine ratio.

Treatment was structured around evidence-based, sequential combination therapy for kidney protection. Empagliflozin 10 mg once daily was switched directly to dapagliflozin 10 mg once daily without overlap. Valsartan/amlodipine 80/5 mg once daily was continued. Liraglutide 0.6 mg once daily was added, with subsequent transition to semaglutide. Finerenone was introduced after albuminuria persisted and serum potassium remained acceptable. Insulin doses were adjusted to improve glycemic control, whereas GLP-1RA doses were titrated upward according to gastrointestinal tolerability. Key follow-up time points and treatment changes are detailed in Table 1 and Figure 1.

By the last follow-up in January 2026, UACR had decreased to 26.53 mg/g Cr and eGFR had increased to 42.48 mL/min/1.73 m^2^. Ordinary least-squares regression of all 10 eGFR measurements from presentation through final follow-up yielded a slope of +4.27 mL/min/1.73 m^2^ per year. Excluding the presentation eGFR of 28.00 mL/min/1.73 m^2^, the slope was +2.08 mL/min/1.73 m^2^ per year. An exploratory analysis of the final eight measurements yielded −1.34 mL/min/1.73 m^2^ per year. A second exploratory analysis using six measurements from the observed eGFR peak onward yielded −3.57 mL/min/1.73 m^2^ per year. All statistical analyses were performed using R version 4.5.2 (R Foundation for Statistical Computing, Vienna, Austria).

During follow-up, HbA1c ranged from 6.8% to 7.9%, and serum potassium ranged from 4.35 to 5.14 mmol/L. Since 2022, blood pressure was generally 110–140/65–90 mm Hg on unchanged valsartan/amlodipine 80/5 mg once daily. BMI remained between 25.2 and 26.2 kg/m^2^. The patient reported good adherence, consistent with outpatient prescription records. The patient reported good overall treatment tolerability and no symptoms suggestive of hypotension, clinically significant volume depletion, urinary tract infection, severe gastrointestinal intolerance, or severe hypoglycemia. No diuretic or nonsteroidal anti-inflammatory drug use or contrast exposure was documented. No severe hypoglycemia, symptomatic hypotension, AKI, clinically significant volume depletion or overload, urinary tract infection, severe gastrointestinal intolerance, clinically significant hyperkalemia requiring treatment interruption, or treatment discontinuation because of adverse events were documented. No hospitalization was documented.

The patient reported that the sequential treatment plan and regular laboratory monitoring were acceptable and expressed willingness to continue long-term therapy. He considered the improvement in his kidney-related laboratory results encouraging.

## 3. Discussion

During sequential kidney-protective therapy with safety monitoring, the eGFR slope over the full follow-up in this patient with T2DM and albuminuric CKD met the recently proposed criterion for clinical remission of CKD [3]. UACR declined overall and remained below 30 mg/g Cr at the last four measurements from May 2025 to January 2026. We retained the full-follow-up slope as the primary descriptive estimate because it included all 10 eGFR measurements without selecting a starting point based on the observed values. The slope remained above the proposed threshold after exclusion of the presentation value. However, the positive full-period slope was driven largely by the early eGFR rise, whereas both later exploratory slopes fell below the threshold. The peak-onward slope may be affected by measurement variability and regression to the mean because it is based on approximately 14 months, starting at the observed maximum. Thus, the slope depends on the assessment period. Longer follow-up is needed to assess the durability of the observed eGFR trajectory.

During follow-up, HbA1c remained within the individualized target range of <6.5% to <8.0% recommended in 2022 Kidney Disease: Improving Global Outcomes (KDIGO) guidelines for non-dialyzed diabetic CKD patients, consistent with the American Diabetes Association (ADA) and KDIGO consensus on personalized targets [8,9]. Blood pressure remained below the individualized target of <140/90 mm Hg for low-risk patients, as recommended in the same consensus [8,9]. Available data showed relatively stable BMI and blood pressure with unchanged antihypertensive therapy, making changes in blood pressure or weight less likely to explain the eGFR and UACR trajectories. However, standardized office blood pressure measurements, volume-status assessments, and objective adherence assessments were unavailable at every visit. Therefore, residual confounding cannot be excluded.

Notably, clinical remission of CKD in patients with T2DM and CKD is a novel proposed concept. The eGFR slope is an established surrogate endpoint for long-term CKD progression and kidney failure, and a chronic eGFR slope more than −1 mL/min/1.73 m^2^ per year has recently been proposed as a threshold for CKD remission [3,10,11]. In this patient, the eGFR slope over the full follow-up met the proposed criterion for clinical remission of CKD during evidence-based therapy with careful monitoring. Although this temporal association does not establish causality, the observed clinical course may enhance clinician confidence in timely treatment intensification and encourage long-term adherence. Importantly, remission should not be equated with cure. Continued therapy, surveillance of kidney function and albuminuria, and monitoring for treatment-related adverse events remain essential.

The treatment strategy reflects the contemporary shift toward integrated cardiorenal protection in T2DM with CKD [8]. RASi therapy was continued for hypertension and albuminuric CKD to reduce intraglomerular pressure and albuminuria [4]. With a decline in eGFR, SGLT2i therapy with empagliflozin was started in April 2022. Empagliflozin was continued until a within-class switch to dapagliflozin at presentation to our clinic in May 2023. Current guidelines recommend SGLT2i as the first-line treatment for patients with T2DM and CKD when eGFR is at least 20 mL/min/1.73 m^2^ [2,8]. SGLT2i restores tubuloglomerular feedback, and retards long-term eGFR decline [12]. Given that the patient was overweight, had concomitant cardiovascular risk factors, and remained at extremely high risk of CKD progression despite SGLT2i therapy, GLP-1RA was subsequently added to improve glycemic control, body weight, albuminuria, and kidney outcomes [6]. GLP-1RA was initiated at a low daily dose and switched to a once-weekly agent for gastrointestinal tolerability [13]. The FLOW trial further supports GLP-1RA therapy as a kidney-protective strategy in high-risk patients with T2DM and CKD [14]. Persistent albuminuria despite RASi therapy provided the rationale for adding finerenone. Guidelines recommend finerenone for patients with T2DM and CKD who have persistent albuminuria despite maximally tolerated RASi therapy, eGFR of at least 25 mL/min/1.73 m^2^, and normal serum potassium [2,8]. Finerenone targets mineralocorticoid receptor-mediated inflammation and fibrosis, reducing albuminuria and kidney events [7]. Combination therapy with finerenone and empagliflozin has shown additive albuminuria-lowering effects [15]. In this case, the finerenone dose was increased based on continuously monitored kidney function and serum potassium levels. A modeling analysis suggests that integrating SGLT2i, GLP-1RA, and finerenone with RASi may further reduce residual cardiorenal-metabolic risk [16]. These complementary mechanisms illustrate the rationale for layered combination kidney-protective therapy.

Safety is an important consideration when combining multiple kidney-protective therapies. In this case, stepwise initiation, dose adjustment, and regular monitoring allowed therapy to be maintained without discontinuation for adverse events. This observation does not negate known risks, including early hemodynamic eGFR changes with SGLT2i, gastrointestinal intolerance with GLP-1RA, or the risk of clinically significant hyperkalemia requiring treatment interruption with finerenone. Rather, it illustrates how sequential implementation may help distinguish expected hemodynamic eGFR changes from disease progression or adverse reactions while reducing the safety concerns of simultaneous multidrug initiation [17,18].

Alternative explanations should be considered. The CKD etiology was likely multifactorial and was not biopsy confirmed. Long-standing T2DM, diabetic retinopathy, persistent albuminuria, and progressive eGFR decline supported diabetes as an important contributor to CKD. No hematuria or other findings suggestive of active urinary sediment were reported, and ultrasonography showed no structural abnormality that explained the decline in kidney function. However, a mixed diabetic and hypertensive etiology could not be excluded [2]. Before presentation, kidney function had declined despite ongoing RASi and SGLT2i therapy. Following a within-class SGLT2i switch and GLP-1RA initiation, a gradual increase in eGFR was observed over time. Following finerenone addition, a modest increase in eGFR was observed, followed by a decrease. UACR declined overall during follow-up, with the last four measurements below 30 mg/g Cr. This temporal sequence is consistent with a potential effect of intensified kidney-protective therapy but does not establish causality. This temporal pattern is consistent with the possibility that targeting inflammatory and fibrotic pathways may complement hemodynamic mechanisms of kidney protection in moderate-to-advanced CKD. However, most of the eGFR gain occurred before finerenone addition. This case also illustrates the feasibility of sequential, evidence-based, remission-oriented therapy.

Real-world reports applying the proposed eGFR slope-based criterion for clinical remission of CKD during sequential combination therapy with the four classes of kidney-protective agents in T2DM and CKD remain limited. This case illustrates a longitudinal outpatient approach: assess residual risk through UACR and eGFR trajectories, add therapies according to indication and tolerability, monitor serum potassium, volume status, and kidney function, and avoid premature discontinuation of treatment in the absence of limiting adverse events. The case also illustrates that remission-oriented care requires longitudinal assessment rather than reliance on isolated eGFR or UACR measurements.

This case has several limitations. The retrospective single-patient design precludes causal inference and may be subject to information bias and residual confounding. The absence of a kidney biopsy precluded histopathologic confirmation of the CKD etiology. Serial eGFR and UACR measurements may have been affected by biological, analytical, and hemodynamic variability, and the pre-presentation decline in eGFR may have included an unrecognized reversible component. The positive full-period slope largely reflected the early eGFR rise, whereas neither later exploratory slope met the proposed criterion. Longer follow-up is needed to assess the long-term trajectories of kidney function and albuminuria. Finally, the proposed eGFR slope-based criterion for clinical remission of CKD has not been externally or prospectively validated.

## 4. Conclusions

In this patient with T2DM and albuminuric CKD, the eGFR slope over the full follow-up met the proposed criterion for clinical remission of CKD during sequential multidrug kidney-protective therapy with safety monitoring. Prospective controlled studies with longer follow-up are needed to evaluate the efficacy and safety of this approach and identify patients who may benefit. The proposed criterion also requires prospective validation against long-term kidney outcomes.

## Figures and Tables

**Table 1:** Serial Laboratory Findings and Treatment Changes During Follow-up.

Date	UACR (mg/g Cr)	eGFR (mL/min/1.73 m^2^)	HbA1c (%)	Serum Potassium (mmol/L)	BMI (kg/m^2^)	Treatment Changes
17 May 2023	121.60	28.00	7.5	4.40	26.2	EMPA discontinued and directly replaced with DAPA 10 mg PO qd; Lira 0.6 mg SC qd started; IDegAsp 16 U SC qd and valsartan/amlodipine 80/5 mg PO qd continued.
13 July 2023	NA	33.77	7.9	NA	NA	No change.
12 March 2024	73.68	43.28	7.8	4.70	25.7	IDegAsp reduced to 14 U SC qd; Lira increased to 1.2 mg SC qd, then switched 2 weeks later to Sema 0.5 mg SC qw; finerenone 10 mg PO qd subsequently started.
27 May 2024	57.28	45.80	7.4	4.35	25.2	IDegAsp increased to 14–18 U SC qd.
19 November 2024	51.58	46.37	7.1	5.14	25.2	Sema increased to 0.75–1.0 mg SC qw; low-potassium diet advised.
18 February 2025	36.12	45.30	6.9	4.68	25.2	Sema increased to 1.0 mg SC qw.
9 May 2025	17.86	43.83	7.9	4.64	NA	IDegAsp increased to 20 U SC qd.
30 July 2025	16.37	43.18	7.8	4.78	25.8	No change.
21 October 2025	26.98	42.59	7.2	4.68	25.6	Finerenone increased to 20 mg PO qd.
9 January 2026	26.53	42.48	6.8	4.64	25.5	No change.

Notes: Empagliflozin was replaced with dapagliflozin due to hospital formulary availability. NA indicates that no test result was available in the hospital information system for that visit. The specific reason for the missing data cannot be determined from the retrospective medical records. Abbreviations: Cr, creatinine; DAPA, dapagliflozin; eGFR, estimated glomerular filtration rate; EMPA, empagliflozin; HbA1c, hemoglobin A1c; IDegAsp, insulin degludec/insulin aspart; Lira, liraglutide; NA, not available; PO, oral administration; qd, once daily; qw, once weekly; SC, subcutaneous administration; Sema, semaglutide; U, units; UACR, urine albumin-to-creatinine ratio.

## Data Availability

All authors confirm that all the data that support the findings of this report are available on request from the corresponding author.

## Abbreviations

The following abbreviations are used in this manuscript:

ADA	American Diabetes Association
AKI	acute kidney injury
BMI	body mass index
CKD	chronic kidney disease
Cr	creatinine
DAPA	dapagliflozin
eGFR	estimated glomerular filtration rate
EMPA	empagliflozin
GLP-1RA	glucagon-like peptide-1 receptor agonist
HbA1c	hemoglobin A1c
IDegAsp	insulin degludec/insulin aspart
KDIGO	Kidney Disease: Improving Global Outcomes
Lira	liraglutide
NA	not available
nsMRA	nonsteroidal mineralocorticoid receptor antagonist
PO	oral administration
qd	once daily
qw	once weekly
RASi	renin–angiotensin system inhibitor
SC	subcutaneous administration
Sema	semaglutide
SGLT2i	sodium–glucose cotransporter 2 inhibitor
T2DM	type 2 diabetes mellitus
U	units
UACR	urine albumin-to-creatinine ratio
